# Disruption of the *Abdominal-B* Promoter Tethering Element Results in a Loss of Long-Range Enhancer-Directed Hox Gene Expression in *Drosophila*

**DOI:** 10.1371/journal.pone.0016283

**Published:** 2011-01-21

**Authors:** Margaret C. W. Ho, Benjamin J. Schiller, Omar S. Akbari, Esther Bae, Robert A. Drewell

**Affiliations:** 1 Biology Department, Harvey Mudd College, Claremont, California, United States of America; 2 College of Osteopathic Medicine of the Pacific, Western University of Health Sciences, Pomona, California, United States of America; Centre for Genomic Regulation (CRG), Universitat Pompeu Fabra, Spain

## Abstract

There are many examples within gene complexes of transcriptional enhancers interacting with only a subset of target promoters. A number of molecular mechanisms including promoter competition, insulators and chromatin looping are thought to play a role in regulating these interactions. At the *Drosophila* bithorax complex (BX-C), the IAB5 enhancer specifically drives gene expression only from the *Abdominal-B* (*Abd-B*) promoter, even though the enhancer and promoter are 55 kb apart and are separated by at least three insulators. In previous studies, we discovered that a 255 bp *cis*-regulatory module, the promoter tethering element (PTE), located 5′ of the *Abd-B* transcriptional start site is able to tether IAB5 to the *Abd-B* promoter in transgenic embryo assays. In this study we examine the functional role of the PTE at the endogenous BX-C using transposon-mediated mutagenesis. Disruption of the PTE by *P* element insertion results in a loss of enhancer-directed *Abd-B* expression during embryonic development and a homeotic transformation of abdominal segments. A partial deletion of the PTE and neighboring upstream genomic sequences by imprecise excision of the *P* element also results in a similar loss of *Abd-B* expression in embryos. These results demonstrate that the PTE is an essential component of the regulatory network at the BX-C and is required *in vivo* to mediate specific long-range enhancer-promoter interactions.

## Introduction

**Figure 1:**
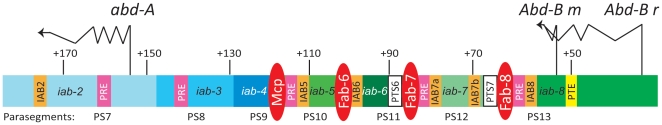
A network of interacting *cis*-regulatory modules control embryonic expression of *abdominal-A* and *Abdominal-B* in the *Drosophila* bithorax complex. The *abd-A* and *Abd-B morphogenetic* (*m*) and *regulatory* (*r*) transcripts are indicated by leftward arrows. The regulatory regions *iab-2*, *iab-3* and *iab-4* (blue) interact with *abd-A*. The *iab-5*, *iab-6*, *iab-7* and *iab-8* regions (green) interact with *Abd-B m*. Each *iab* region is thought to contain at least one characterized enhancer (orange rectangles) capable of directing Hox gene expression in a specific embryonic parasegments (PS). The positions of the characterized Fab-6, Fab-7, Fab-8 and Mcp insulators (red ellipses), promoter targeting sequence (PTS) modules (white rectangles), promoter tethering element (PTE) (yellow rectangle) and polycomb response elements (PREs) (pink rectangles) are indicated. Numbers above line refer to kilobase positions in DNA sequence accession number: DM31961.

To ensure a high fidelity of gene expression patterns in embryos a very strict functional requirement exists for the interaction of *cis*-regulatory modules (CRMs) in the genome of animals during development [1], [2], [3], [4]. Embryonic transcriptional enhancers, which direct specific spatio-temporal patterns of gene expression in the embryo, are not permitted to promiscuously activate transcription from non-target promoters. Chromatin structural organization, insulators and promoter competition are thought to play a role in regulating enhancer-promoter interactions ([5], [6], [7], [8], [9], for recent reviews see [10], [11], [12]). At the *Drosophila* bithorax complex (BX-C), an extensive network of CRMs located in over 300 kb of *infraabdominal* (*iab*) non-genic sequence is responsible for directing embryonic expression of just three homeotic genes; *Ultrabithorax* (*Ubx*), *abdominal-A* (*abd-A*) and *Abdominal-B* (*Abd-B*) (Fig. 1) [13], [14]. These three homeotic genes are critical for establishing cellular identities in the presumptive thoracic and abdominal segments during development [2], [15].

We recently identified a 255 bp promoter tethering element (PTE) located 5′ of the *Abd-B* transcriptional start site that is responsible for specifically recruiting the IAB5 enhancer from the BX-C to the *Abd-B* promoter in competition assays on transgenes [16]. Furthermore, the PTE also demonstrates anti-insulator activity by enabling the IAB5 enhancer to bypass an insulator from the BX-C to activate a target promoter on transgenes [17]. The ability of the PTE to facilitate specific enhancer-promoter interactions may explain how certain IAB enhancers, such as IAB5, IAB6, IAB7a and IAB7b [8], [18], [19], are able to bypass the *Frontabdominal* (*Fab*) insulators Fab-6, Fab-7 and Fab-8 [19], [20], [21], [22] and direct transcription from the *Abd-B morphogenetic* (*m*) promoter at the endogenous BX-C (Fig. 1). As such, the PTE may be part of the complex regulatory network of CRMs, including the insulators, Promoter Targeting Sequences (PTSs) [23], [24] and additional tethering sequences located 5′ of the *Abd-B* gene [25], [26], responsible for mediating enhancer-promoter interactions in the BX-C. In contrast to the PTSs, which are located adjacent to the Fab-7 and Fab-8 insulators, the PTE is adjacent to the *Abd-B* promoter (Fig. 1). The PTSs have been implicated in mediating insulator bypass for the IAB enhancers on transgenic constructs [23], [24]. However, deletion of either of the characterized PTSs from the endogenous BX-C did not result in a significant phenotype [8], [18], [19], suggesting that there might be other sequences capable of mediating the long-range enhancer-promoter specificity at the BX-C.

In this study, we demonstrate the functional importance of the PTE at the endogenous BX-C. Disruption of the PTE by a *P* element insertion *in vivo* results in a loss of IAB enhancer-directed *Abd-B* expression during development and a homeotic phenotype when placed in hemizygosity with an *Abd-B* null allele. Further molecular analysis of a mutant generated by imprecise excision of the *P* element reveals that a partial deletion of the PTE and neighboring upstream genomic sequences results in a similar loss of enhancer-directed *Abd-B* expression. These results demonstrate the critical functional role that the PTE plays in regulating promoter-enhancer interactions at the endogenous *Drosophila* BX-C.

## Results

### Insertions at the PTE disrupt *Abd-B* expression and result in homeotic transformation

**Figure 2:**
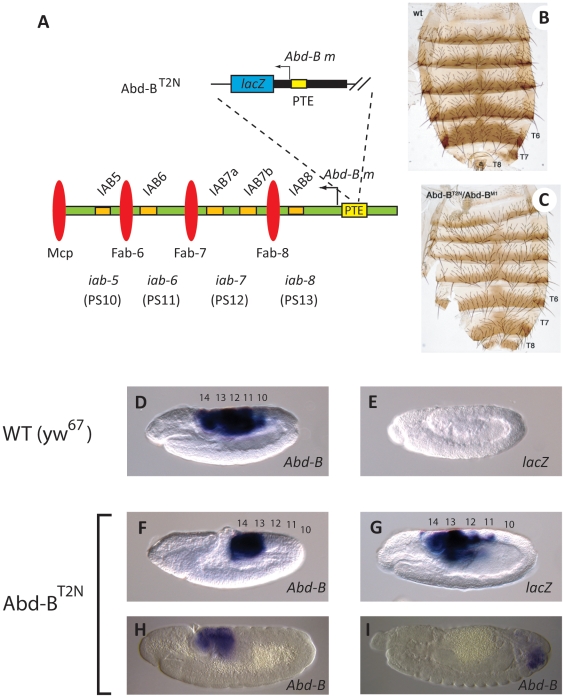
*P* element insertion into the endogenous PTE results in homeotic transformation and loss of *Abd-B* expression. (**A**) Schematic diagram of the *Abd-B^T2N^ P* element line, showing the insertion site −241 bp 5′ of the *Abd-B m* transcription start site in the endogenous PTE sequence. The same symbols and color scheme as in Fig. 1 indicate the neighboring *cis*-regulatory modules. (**B**) Dorsal cuticles were prepared from adult females. A normal abdominal pigmentation pattern is observed in wild-type (WT) adults. (**C**) In *Abd-B^T2N^*/*Abd-B^M1^* hemizygotes the seventh abdominal tergite (T7) is fully developed, indicating a transformation toward a more anterior abdominal segment identity. The eighth abdominal tergite (T8) also shows a partial transformation toward a more anterior identity. *Abd-B^M1^* is a Class III null allele for both the *Abd-B m* and *r* transcripts [15], [27]. *Abd-B* gene expression pattern in wild-type (WT) (**D**) and *Abd-B^T2N^* (**F**) germ-band elongation stage 9 embryos. The staining patterns for the *Abd-B^T2N^* line show reduced *Abd-B* expression in parasegments (PS) 10, 11 and 12 at stage 9 (**F**), stage 11 (**H**) and stage 13 (**I**) of development. The *lacZ* reporter gene is expressed in developing posterior regions of the *Abd-B^T2N^* embryos (**G**). Expression is strongest in PS13, although the pattern extends from PS 10–14 and is very similar to the endogenous *Abd-B* transcription pattern. No *lacZ* expression is detectable in WT embryos (**E**).

Genetic studies were carried out to determine whether the activity of the PTE is important for *in vivo* IAB enhancer–*Abd-B* interactions in the context of the BX-C. The previously identified *Abd-B^T2N^* mutation [27] is derived from a *P* element insertion in the endogenous PTE sequence (−241 bp relative the *Abd-B* transcription start site). The original *P* element insertion line (*Abd-B^Lac1^*) contains a 5.6 kb *Abd-B* promoter region which includes 4.3 kb of 5′ sequence harboring the PTE sequence, fused to a *lacZ* reporter gene. This *P* element also contains the *Fab-8* insulator element, an IAB8 enhancer element and the *rosy* gene. In contrast, the derived *Abd-B^T2N^* line contains only a truncated *Abd-B* promoter-*lacZ* reporter gene, generated by imprecise excision of the 5′ region of the *P* element (Fig. 2a). In the *Abd-B^T2N^* line the *P* element is inserted within the endogenous 255 bp PTE sequence, while leaving the entire *Abd-B morphogenetic* (*m*) transcription unit intact [27]. The *Abd-B^T2N^* mutant is described as a Class I *Abd-B* mutant, capable of complementing mutations in the *Abd-B regulatory* (*r*) transcript, but affecting *Abd-B m* transcript expression [27]. In hemizygosis with the *Abd-B^M1^* null allele, the *Abd-B^T2N^* insertion line shows a phenotypic transformation of abdominal segments 5–8 towards a more anterior abdominal segment identity. This is seen most clearly in the complete transformation of the seventh abdominal tergite to a more anterior identity in the *Abd-B^T2N^* insertion line (Fig. 2c) when compared to wild-type (WT) (Fig. 2b). The homeotic transformation is easiest to see in the cuticles of adult females due to their lighter abdominal pigmentation when compared to males (Fig. 2b and c), although it is apparent in both sexes. This phenotype is characteristic of *Abd-B m* mutations [27], [28], suggesting that the *P* element insertion into the PTE is disrupting expression from the adjacent *Abd-B m* promoter and is consistent with a reduction in IAB5-7 enhancer-*Abd-B* interactions.

In WT *D. melanogaster* embryos, the expression pattern of the *Abd-B m* transcript extends from parasegment (PS) 10–13 during the germ-band elongation stage of development, while the *r* transcript is predominantly restricted to PS14 at this stage [29], [30]. *In situ* hybridization with an RNA probe that detects both the *m* and *r* transcripts in germ-band elongation stage embryos generated from crosses of heterozygous balanced *Abd-B^T2N^* adults (Fig. 2f) [27], demonstrates a loss of *Abd-B* expression in PS10–12 (presumptive abdominal segments 5–7) when compared with expression in WT embryos (Fig. 2d). These results indicate that the transposon insertion in the PTE impairs the ability of the IAB5–7 enhancers in the BX-C to drive expression from the endogenous *Abd-B m* promoter. The detectable expression of *Abd-B* in PS13 in *Abd-B^T2N^* embryos raises the question of why there is a homeotic transformation of the eighth abdominal segment in hemizygous *Abd-B^T2N^*/*Abd-B^M1^* adults. While this phenotype is significant, it is only a partial transformation of A8 towards a more anterior identity (Fig. 2c), suggesting that there is not a complete loss of *Abd-B* patterning function. It is possible that there may be subtle modulation of the level of *Abd-B* gene expression in PS13 which, while responsible for the phenotypic transformation, is not readily detectable using RNA *in situ hybridization*. The expression of *Abd-B* persists in PS13 and 14 at least through stage 13 of development (Fig. 2h and i). However, it is also possible that transcription of *Abd-B* in late embryonic or larval stages may be modulated from the *Abd-B^T2N^* allele.

Knowing that expression from the endogenous *Abd-B m* promoter was perturbed in *Abd-B^T2N^* mutants, we examined whether the enhancers from the BX-C were re-directed to the intact ectopic *Abd-B m* promoter (containing the PTE sequence), which drives *lacZ*, on the *P* element insertion. In *Abd-B^T2N^* embryos, *lacZ* expression was detected in a pattern extending from PS10 to 14 (Fig. 2g). The *lacZ* expression appears strongest in PS13 (presumptive abdominal segment 8) and weaker in PS10 (Fig. 2g), presumably due to the proximity of the endogenous IAB8 regulatory sequences to the target promoter, as previously observed [27]. This pattern of *lacZ* expression is consistent with the *Abd-B* expression pattern detected in wild-type embryos (Fig. 2d) and indicates that the IAB enhancers from the endogenous BX-C are now being re-directed to the intact ectopic *Abd-B* promoter (containing the PTE) on the *P* element to drive expression of *lacZ* in *Abd-B^T2N^* embryos.

### Deletion of PTE and neighboring upstream sequences results in a loss of enhancer-directed *Abd-B* transcription

**Figure 3:**
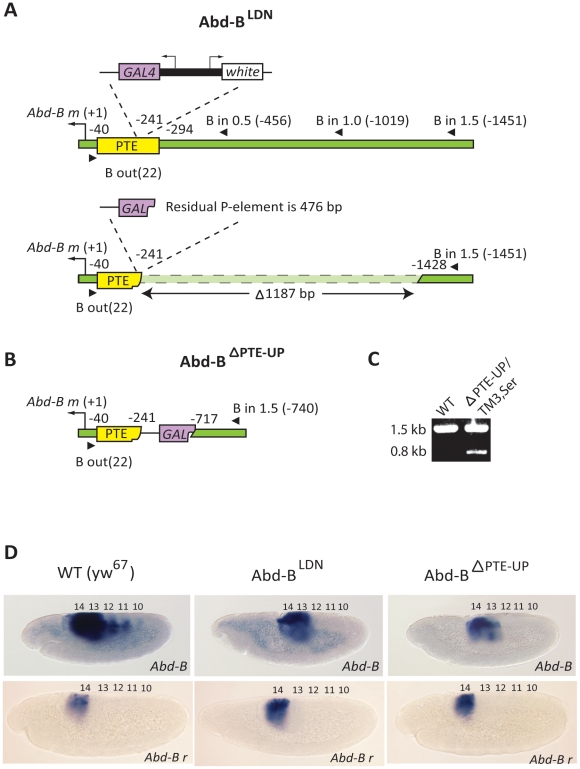
Deletion of the PTE and upstream sequences at the endogenous BX-C disrupts *Abd-B* expression. (**A**) The *Abd-B^LDN^* mutant features a *P* element inserted within the PTE (yellow box) (−241 bp relative to the *Abd-B* transcription start site (black arrow)). The *P* element contains *GAL4* (purple box) and *white* (white box) reporter genes. PCR primers (black triangles) designed against the *Abd-B* promoter region and 0.5 kb, 1 kb and 1.5 kb upstream of the *Abd-B m* transcription start site were used to characterize the approximately 1.2 kb deletion (dashed box) in the *Abd-B* ^ΔPTE-UP^ mutant generated by imprecise excision of the *P* element. (**B**) The resulting *Abd-B* ^ΔPTE-UP^ deletion removes 53 bp of the PTE sequence 5′ of the original insertion site and an additional 1134 bp of endogenous sequence 5′ of the PTE. A 476 bp portion of the *GAL4* coding sequence (purple polygon) from the original *P* element remains, as well as 202 bp of the 3′ PTE sequence (yellow polygon). (**C**) Primers designed against the *Abd-B* promoter region and 1.5 kb upstream of the *Abd-B* transcription start site were used to genotype adult flies carrying the deletion allele by amplifying the 1.5 kb wild-type (WT) band and the 0.8 kb *Abd-B* ^ΔPTE-UP^ band in balanced *Abd-B* ^ΔPTE-UP^ (ΔPTE-UP/TM3,Ser) individuals. (**D**) *In situ* hybridization with an RNA probe that can detect both the *Abd-B m* and *r* transcripts (*Abd-B*) shows WT expression in parasegments (PS) 10–14 in yw^67^ embryos, while expression is found only in PS13–14 in a portion of embryos collected from crosses of balanced *Abd-B* ^ΔPTE-UP^ and *Abd-B* ^LDN^ lines. *In situ* hybridization with a probe specifically designed against the *Abd-B r* transcript (*Abd-B r*) detects identical patterns of expression in PS14 of germ-band elongation stage embryos collected from WT, mutant *Abd-B^LDN^* and *Abd-B^ΔPTE-UP^* balanced lines.

In the *Abd-B^T2N^* mutant endogenous PTE function is disrupted by *P* element insertion. In order to address the loss-of-functional activity of the PTE more directly we generated a mutant line in which the 5′ portion of the PTE and neighboring upstream genomic sequences are deleted from the endogenous BX-C locus (Fig. 3). This was accomplished by performing an imprecise excision of the *P* element insertion from the *Abd-B^LDN^* mutant (Fig. S1). The *Abd-B^LDN^* mutant was generated by a *P* element replacement strategy and carries an insertion containing the *GAL4* and *white* genes in the identical location within the PTE (−241 bp relative to the *Abd-B* transcription start site) as the *Abd-B^T2N^* line, but does not contain an ectopic copy of the PTE (Fig. 3a) [31]. In the *Abd-B^LDN^* line, both the *white* and *GAL4* genes are only expressed clearly in PS13 and very weakly in PS14 in embryos (data not shown), indicating that the distantly located IAB5–7 enhancers in the BX-C are not activating transcription of either reporter gene. The *Abd-B^LDN^* line was utilized to generate an imprecise excision mutant as it carries the readily detectable *white* reporter gene (Fig. 3a). As a result, adult flies in which *P* element excision had occurred were easily identified by loss of red eye color (Fig. S1). PCR-based screening with primers from the proximal *Abd-B m* promoter region and from 0.5 kb, 1 kb and 1.5 kb 5′ of the *Abd-B* transcription start site was used to identify a 1.2 kb deletion in the *Abd-B* ^ΔPTE-UP^ allele (Fig. 3a–c). The molecular nature of the deletion was characterized by sequencing and found to have removed 53 bp of the PTE sequence 5′ of the original *P* element insertion site, as well as 1134 bp of endogenous genomic sequence 5′ of the defined PTE. In this *Abd-B* ^ΔPTE-UP^ allele, a portion of the *GAL4* gene from the original *P* element remains, along with 202 bp of the 3′ PTE sequence (Fig. 3b).

Based on the *Abd-B* expression observed in the *Abd-B^T2N^* insertion line, we hypothesized that the expression pattern of the endogenous *Abd-B* gene from the *Abd-B* ^ΔPTE-UP^ allele may also be perturbed. *In situ* hybridization with an RNA probe that can detect both the *m* and *r* transcripts shows that *Abd-B* gene expression is lost specifically in PS10-12, but not in PS13–14 in germ-band elongation stage embryos generated from crosses of heterozygous balanced *Abd-B* ^ΔPTE-UP^ adults (Fig. 3d). Statistical analysis demonstrates that the number of embryos demonstrating this restricted *Abd-B* expression pattern from both the *Abd-B* ^ΔPTE-UP^ and *Abd-B^LDN^* alleles is highly significant (p<0.01) when compared to the number of embryos with the WT *Abd-B* expression pattern. This restricted pattern of *Abd-B* expression persists at least through stage 13 of development (data not shown). One possible explanation for the loss of *Abd-B* expression in PS10–12 is that the genomic region deleted in the *Abd-B* ^ΔPTE-UP^ allele harbors a CRM capable of driving transcription in these specific parasegments. However, when tested in a transgenic reporter gene assay the 1.2 kb region does not exhibit embryonic enhancer activity (data not shown).

To confirm that mutations in the PTE only affect expression from the *Abd-B m* promoter, but not the *Abd-B r* promoter, *in situ* hybridization with a RNA probe (BPP, [32]) that specifically detects only the *Abd-B r* transcript was performed on embryos collected from WT, *Abd-B^LDN^* and *Abd-B* ^ΔPTE-UP^ balanced lines. The expression pattern of the *Abd-B r* transcript was confirmed to be identical in the WT, *Abd-B^LDN^* and *Abd-B* ^ΔPTE-UP^ embryos, appearing only in PS14 of germ-band elongation stage embryos (Fig. 3d). These observations are consistent with the known pattern of expression from the *Abd-B r* promoter [29] and confirm that the disruption to the PTE in these mutants only affects enhancer-mediated transcription from the *Abd-B m* promoter (see discussion for more detail).

## Discussion

### Parasegment-specific interactions between the IAB enhancers and *Abd-B* promoter in the BX-C

**Figure 4:**
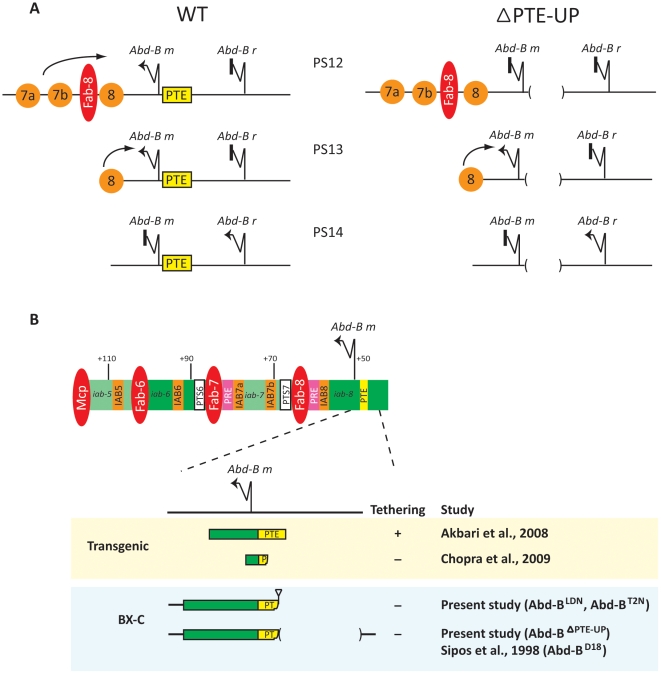
PTE sequence is required for specific promoter-enhancer tethering at the *Abd-B* gene. **(A)** ***Abd-B*** **transcription is disrupted in PTE mutants.** In wild-type (WT) germ-band elongation stage embryos expression from the *Abd-B m* promoter is observed in parasegments (PS) 10–13 and from the *Abd-B r* promoter in PS14 [29]. The loss of *Abd-B* expression in PS10–12 in *Abd-B^ΔPTE-UP^* (ΔPTE-UP) embryos is consistent with a disruption of expression from the *Abd-B m* promoter. In PS12 of WT embryos, the tethering sequences upstream of the *Abd-B m* promoter enable the IAB7a and IAB7b enhancers (7a and 7b, orange circles) to act over the long-range of intervening sequence and bypass the Fab-8 chromatin insulator (red ellipse) and drive expression from the *Abd-B m* promoter. In PS12 of *Abd-B^ΔPTE-UP^* mutants the removal of the tethering sequences disrupts the ability of the IAB7 enhancers to activate the *Abd-B m* promoter across the intervening Fab-8 insulator. A similar disruption of promoter-enhancer tethering activity at *Abd-B^ΔPTE-UP^* is also seen in PS11 (IAB6 enhancer) and PS10 (IAB5 enhancer). In PS13 of both WT and *Abd-B^ΔPTE-UP^* embryos, IAB8 (8, orange circle) is able, due to its physical proximity and lack of intervening chromatin insulator, to drive expression from the *Abd-B m* promoter. In PS14 of both WT and *Abd-B^ΔPTE-UP^* embryos, *Abd-B r* is transcribed independently of IAB embryonic enhancer activity and represses transcription from the *Abd-B m* promoter [28]. **(B) Functional dissection of critical** ***Abd-B*** **tethering sequences.** Schematic diagram of the regulatory region of the *Abdominal-B* gene shows enhancers (orange boxes) in the infraabdominal regions *iab-5* to *iab-8* (green) that are responsible for activating expression of the *Abd-B* gene in abdominal PS 10–13 during embryogenesis. Color scheme for other types of *cis*-regulatory modules is same as in Figure 1. Transgenic studies (highlighted in yellow box) show that a 1.4 kb of sequence from +1228 to −294, (including the 255 bp PTE sequence located 40 bp 5′ of the *Abd-B m* transcription start site), is sufficient for promoter-enhancer tethering [16]. Additional transgenic studies have shown that an approximately 200 bp sequence spanning the *Abd-B m* transcription start site (+100 to −100) is not sufficient for tethering activity [36]. *P* element insertion at the endogenous BX-C (as in the *Abd-B^LDN^* or *Abd-B^T2N^* mutations) demonstrates that the 242 bp sequence 5′ of the *Abd-B m* transcription site (from +1 to −241), including 202 bp of the identified PTE sequence, is not sufficient to tether the distally located IAB5, 6 and 7 enhancers *in vivo*. Deletion of an approximately 1.2 kb sequence (−241 to −1428) which include 53 bp of the 5′ region of the PTE and neighboring upstream sequences in the *Abd-B* ^ΔPTE-UP^ mutant also results in a loss of tethering. The 53 bp region of the PTE may therefore be important for functional tethering. A potential tethering role at the endogenous BX-C for the sequences 5′ of the PTE is also suggested by earlier genetic complementation studies in the *Abd-B^D18^* allele [25].

The absence of *Abd-B* expression in PS10–12 of *Abd-B^ΔPTE-UP^* germ-band elongation stage embryos is consistent with a loss of IAB-enhancer directed expression from the *Abd-B m* promoter [29]. This suggests that deletion of the *Abd-B* promoter tethering sequence (PTE) and the neighboring 1.1 kb 5′ sequence in the *Abd-B^ΔPTE-UP^* line leads to a disruption of the long-range interactions between the *Abd-B m* promoter and enhancers from the *iab-5, iab-6* and *iab-7* regions in PS10, 11 and 12, respectively. For example, in PS12 of WT embryos the tethering sequences upstream of the *Abd-B m* promoter enable the IAB7a and IAB7b embryonic enhancers to bypass the Fab-8 chromatin insulator and drive expression from the *Abd-B m* promoter (Fig. 4a). In *Abd-B^ΔPTE-UP^* mutant embryos, removal of the tethering sequences appears to disrupt the ability of the IAB7a and IAB7b enhancers to activate the *Abd-B m* promoter, resulting in an absence of *Abd-B* expression in PS12 (Fig. 4a and 3d). Similarly, the IAB6 and IAB5 enhancers are unable to bypass intervening insulators to activate *Abd-B m* expression in PS11 and PS10 in *Abd-B^ΔPTE-UP^* embryos. In contrast to PS10–12, the specific enhancer-promoter interactions at the BX-C in PS13 appear to be intact in *Abd-B^ΔPTE-UP^* embryos. In WT embryos the IAB8 embryonic enhancer, located 3′ of the *Abd-B* gene, is solely responsible for directing expression from the *Abd-B m* promoter in PS13 [33]. In *Abd-B^ΔPTE-UP^* mutant embryos the *Abd-B m* transcript remains strongly expressed in PS13 (Fig. 3d). This result indicates that the interaction between the IAB8 enhancer and the *Abd-B m* promoter may not require tethering activity, likely due to the physical proximity of the enhancer to the promoter and lack of an intervening chromatin insulator (Fig. 4a). However, given the partial transformation phenotype observed for PS13 in flies carrying a disruption of the PTE sequences (as in the case of the *Abd-B^T2N^* allele, Fig. 2c) it remains possible that loss of PTE activity may be responsible for subtle effects on transcription of *Abd-B* in PS13. In PS14, *Abd-B* expression in germ-band elongation stage embryos is not driven by the 3′ IAB enhancers, as it is initiated from the *r* transcriptional start site located 5′ of the PTE sequence (Fig. 1) [29], [34]. Consequently, expression of the *r* transcript in PS14 is not perturbed by the loss of tethering activity in *Abd-B^ΔPTE-UP^* mutant embryos (Fig. 4a and 3d).

One possible outcome of the disruption of IAB enhancer interactions with the *Abd-B m* promoter at the *Abd-B^ΔPTE-UP^* allele may be the re-direction of those enhancers to the neighboring *abd-A* promoter in the BX-C. However, no change in the expression pattern of *abd-A* (extending from PS7 to PS13 in germ-band elongation stage embryos [35] could be detected from the *Abd-B^ΔPTE-UP^* or *Abd-B* ^LDN^ alleles (data not shown).

### Functional dissection of critical sequences at the PTE

The functional activity of the PTE at the endogenous BX-C prompts the question of exactly what sequences associated with the PTE are necessary to confer promoter-enhancer tethering. Our earlier studies demonstrated that a 255 bp region located between −40 and −294 bp 5′ of the *Abd-B m* transcription start site is sufficient to tether the IAB5 enhancer to an ectopic promoter in a transgene competition assay (Fig. 4b) [16]. In contrast, an approximately 200 bp region extending from −100 to +100 relative to the *Abd-B m* transcriptional start site is not sufficient to mediate promoter-enhancer tethering when tested in similar transgenic assays [36]. In agreement with this observation, the 202 bp of sequence from the 3′ end of the PTE remaining at the endogenous BX-C in the *Abd-B^ΔPTE-UP^* allele is also not sufficient to mediate tethering of the IAB enhancers to the *Abd-B m* promoter (Fig. 4b). A previous study by Sipos and colleagues, utilizing an *Abd-B* mutant allele harboring a small deletion at the *Abd-B m* promoter region and a deletion at the *iab-7* region carried on opposite chromosomes resulted in *Abd-B* transcriptional activity [25]. This indicates that a *trans* interaction can occur between the *iab-7* regulatory region and the remaining *Abd-B* promoter region across chromosomes. The same genetic complementation test using the *iab-7* mutant allele and the *Abd-B^D18^* deletion allele (which removes approximately 8 kb of sequence 5′ of the *Abd-B m* transcription start site) on opposite chromosomes showes an increase in *trans*-mediated *Abd-B* activity [25]. These results indicate that additional sequences located 5′ of the 255 bp PTE may also contribute to tethering activity. A functional role for the sequences in the 5′ end of the PTE and neighboring upstream genomic regions is supported by the loss of enhancer-directed *Abd-B m* transcript expression at the *Abd-B^ΔPTE-UP^* allele after deletion of the 5′ 53 bp region in the PTE and 1134 bp of sequence located 5′ of the defined PTE (Fig. 4b). The loss of *Abd-B* expression observed in the *Abd-B^ΔPTE-UP^* mutant is consistent with the previously reported molecular phenotype of *Abd-B m* mutants [27] and confirms the role of the PTE and associated 5′ sequences in mediating specific IAB enhancer-directed expression of the *Abd-B m* transcript.

### Promoter tethering as a general regulatory mechanism

Additional examples of genetic complexes in which long-range interactions between CRMs have been characterized include the human beta-globin locus [37], the *Drosophila Antennapedia* complex [38], [39] and mouse *HoxD* complex [3]. In the large genomes of vertebrates, where extensive global control regions have been identified, promoter-enhancer tethering is emerging as a critical general mechanism for regulation of gene expression [3], [37]. More recently, in *Drosophila*, other PTE sequences have been located in the *even-skipped* [40] and *engrailed* [41] loci that mediate enhancer-promoter communication. The long-range CRM communication in these critical developmental genes has been shown to be dynamic, changing through the course of development in some cases [40] and perhaps even capable of mediating the evolution of novel patterns of gene expression in different insect species [42].

Our current model for the molecular function of the PTE in the *Drosophila* BX-C is that regulatory interactions that enable the PTE to tether the IAB enhancers to the *Abd-B m* promoter in specific parasegments during embryonic development may be mediated by chromatin looping [17]. A number of studies have indicated that chromatin looping may facilitate promoter-enhancer tethering through the action of different transcription factors. For example, the sea urchin GCF1 protein is able to form higher order multimeric loop structures when added to target site oligonucleotides *in vitro* [43]. The abundant mammalian transcription factor Sp1 has also been shown to form multimers and to strongly facilitate *in vivo* activation of a promoter by distantly located enhancer CRMs [44], [45]. More recently, molecular studies have used high-magnification confocal imaging and 2D RNA fluorescence *in situ* hybridization (FISH) to visualize specific physical associations between distantly located *cis*-regulatory sequences in the nucleus at the human beta-globin locus [37].

Direct evidence for chromatin looping at the *Drosophila* BX-C comes from a study examining physical chromosomal interactions using probes designed against the IAB5 and IAB8 enhancers and a promoter-proximal region upstream of the *Abd-B m* transcription unit, containing the PTE sequence [46]. In a portion of nuclei taken from the eighth abdominal segment region of a germ-band elongation stage embryo, FISH signals from the *Abd-B m* promoter region co-localize with signals from the distal IAB5 enhancer, while the more proximal IAB8 enhancer remains distantly located and disassociated [46]. This result suggests that physical associations may indeed occur between the IAB5 enhancer and the 5′ upstream region of the *Abd-B m* promoter to facilitate expression of the *Abd-B m* transcript. These data support our model that tethering mediates the interaction of long-range enhancers (such as IAB5) to the *Abd-B m* promoter, but that it is not required for the functional interaction of the 3′ proximal IAB8 enhancer to the *Abd-B m* promoter.

In our current model the PTE may bind sequence-specific protein factors which interact with complementary factors bound near the IAB enhancers, allowing a molecular bridge or loop to form between the *Abd-B m* promoter and the IAB enhancers [17]. This model does not exclude the possibility that additional molecular interactions between known regulatory regions in the BX-C, including insulator and PTS sequences, may also mediate enhancer-promoter communication. In future studies other techniques, such as three-dimensional chromatin conformation capture (3C) [47] and *Dam* methylase identification [48], will be well suited to more fully elucidate the nature of such interactions and the molecular mechanisms of PTE function. In addition, molecular dissection of the functional sequences within the PTE and 5′ associated sequences in transgenic tethering assays and the biochemical identification of putative sequence-specific DNA-binding *trans* factors that may mediate the PTE activity will be essential.

## Materials and Methods

### Genetic insertion lines

The *Abd-B^T2N^* and *Abd-B^LDN^ P* element fly lines inserted −241 bp 5′ of the *Abd-B* m transcription start site were provided by Ernesto Sanchez-Herrero [27], [31].

### *P* element imprecise excision from the *Abd-B^LDN^* line

*Abd-B^LDN^* flies were crossed with a transposase expressing line (Δ2–3) carrying a Stubble (Sb) dominant phenotypic marker (BL Stock 1798) in Cross 1 (Fig. S1). Cross 1 male progeny were screened for Sb (marking the presence of Δ2–3 transposase) and variegated red eye color (rather than white eye color). The selected Cross 1 males were crossed with female with D, a dominant marker, and a TM3 balancer chromosome carrying Serrate (Ser), a dominant phenotypic marker (BL Stock 7198) in Cross 2 (Fig. S1). The male Cross 2 progeny were screened for Ser (marking the presence of the TM3 balancer), white eyes (indicating excision of the *Abd-B*-*GAL4^LDN^ P* element construct), and the absence of Sb (indicating loss of the Δ2–3 transposase) as well as against D. The selected Cross 2 male flies were crossed with BL Stock 7198 flies again in Cross 3 (Fig. S1). After a few days, the male parents were recovered from Cross 3 vials. PCR amplification of the Abd-B region with primers located 0.5 kb, 1 kb, and 1.5 kb upstream of the *Abd-B* transcription start site on the genomic DNA prepared from approximately 100 selected Cross 2 male flies was used to detect deletions in the PTE sequence.

Primers used:

*Abd-B* promoter out: 5′-CGA CAA CAT ATC CAC ATC GCT-3′

*Abd-B* promoter 0.5 kb in: 5′-AAG TGC GAT ACC ATC TTT-3′

*Abd-B* promoter 1 kb in: 5′-TGC CTT TGG AAG TGA GAC AA-3′

*Abd-B* promoter 1.5 kb in: 5′-GGA AAT AGA TTG CGG CAG TTA A-3′

The PCR products were ligated into pGEM-T Easy vector (Promega) and sequenced using T3 and SP6 sequencing primers. Progeny from Cross 3 vials seeded with a male parent exhibiting a disruption of the PTE were screened against D and for Ser and self-crossed to generate a balanced mutant line.

### *In situ* analysis of *abd-A* and *Abd-B* expression

*In situ* hybridization probes to detect transcription of *Abd-B* and *abd-A* were PCR-amplified using *D. melanogaster yw^67^* adult genomic DNA as a template. The previously described DNA sequences for the Bexon region (exon 8 of the *D. melanogaster Abd-B* gene), BPP (specific to the *r* transcript) and Aexon region [32] were PCR amplified and cloned into pGEMT-Easy (Promega). PCR primer sequences were as follows:

Bexon s: 5′-GAACAAGAAGAACTCACAGC-3′;

Bexon as: 5′-TAGGCATAGGTGTAGGTGTAGG-3′;

BPP s: 5′-TATTATTCGTCTCCAGTCGC-3′;

BPP as: 5′-CTCAGATTGATGGTGGTGGTGG-3′;

Aexon s: 5′- CACCAACAGCAGCAACAACAGC-3′ (173566);

Aexon as: 5′- CATTGTATTCAAGCGTTGGC-3′ (174756);

Antisense RNA probes (relative to the direction of *Abd-B* and *abd-A* transcription) were prepared using a digoxigenin (DIG) RNA-labeling kit (Roche, Gipf-Oberfrick, Switzerland). Embryos from each of the wild-type *D. melanogaster,* and mutant *Abd-B^T2N^*, *Abd-B^LDN^* and *Abd-B^ΔPTE-UP^* lines were collected, fixed and hybridized with the appropriate probes as previously described [32]. Anti-sense Bexon RNA probes enable the detection of both the *m* and *r* transcript and anti-sense BPP RNA probes enable the specific detection of only the *Abd-B r* transcript [32].

## Supporting Information

Figure S1 **Crosses to generate Abd-B^ΔPTE-UP^ mutant.** (**A**) Schematic diagram of the *Abd-B^LDN^ P* element insertion line, showing the insertion site −241 bp 5′ of the *Abd-B m* transcription start site in the endogenous PTE sequence. The *P* element insertion in the *Abd-B^LDN^* line contains *GAL4* (purple box) and *white* (white box) reporter genes. The same symbols and color scheme as shown in Fig. 1 are used to show the *cis*-regulatory modules. (**B**) *Abd-B^LDN^* flies were crossed with a transposase expressing line (Δ2–3) carrying Stubble (Sb) (BL Stock 1798) (Cross 1). Cross 1 male progeny were screened for Sb and variegated red eye color (rather than white eye color). (**C**) The selected Cross 1 males were crossed with a female with D, a dominant marker, and a TM3 balancer chromosome carrying Serrate (Ser), a dominant phenotypic marker (BL Stock 7198) (Cross 2). Cross 2 male progeny were screened for Ser, white eyes (indicating excision of the *GAL4 LDN P* element construct), and the absence of Sb and D. (**D**) The selected Cross 2 male flies were crossed with BL Stock 7198 flies again (Cross 3). After a few days, the male parents were recovered from the Cross 3 vials. PCR amplification of the *Abd-B* promoter region with primers (black triangles) located 0.5 kb, 1 kb, and 1.5 kb upstream of the *Abd-B* transcription start site on the genomic DNA prepared from these selected Cross 2 male flies was used to detect deletions in the PTE sequence. Progeny originating from Cross 3 vials seeded with a male parent exhibiting a disruption of the PTE (ΔPTE) were recovered and screened against D and for Ser. (**E**) These selected Cross 3 progeny were then self-crossed to generate the *Abd-B* ^ΔPTE-UP^/TM3,Ser balanced line.
(TIF)
