# Correction: Disruption of the *Abdominal-B* Promoter Tethering Element Results in a Loss of Long-Range Enhancer-Directed Hox Gene Expression in *Drosophila*

**DOI:** 10.1371/annotation/613079ca-aeae-4d29-bc5c-aa7202e6993e

**Published:** 2011-02-09

**Authors:** Margaret C. W. Ho, Benjamin J. Schiller, Omar S. Akbari, Esther Bae, Robert A. Drewell

Equal contribution was unassigned. Margaret C. W. Ho and Benjamin J. Schiller contributed equally to this work.

