# Supplementary figures and images for: Disruption of the Abdominal-B Promoter Tethering Element Results in a Loss of Long-Range Enhancer-Directed Hox Gene Expression in Drosophila
Source: PLoS One. 2011 Jan 21;6(1):e16283. doi: 10.1371/journal.pone.0016283 (PMC3025016; doi:10.1371/journal.pone.0016283)

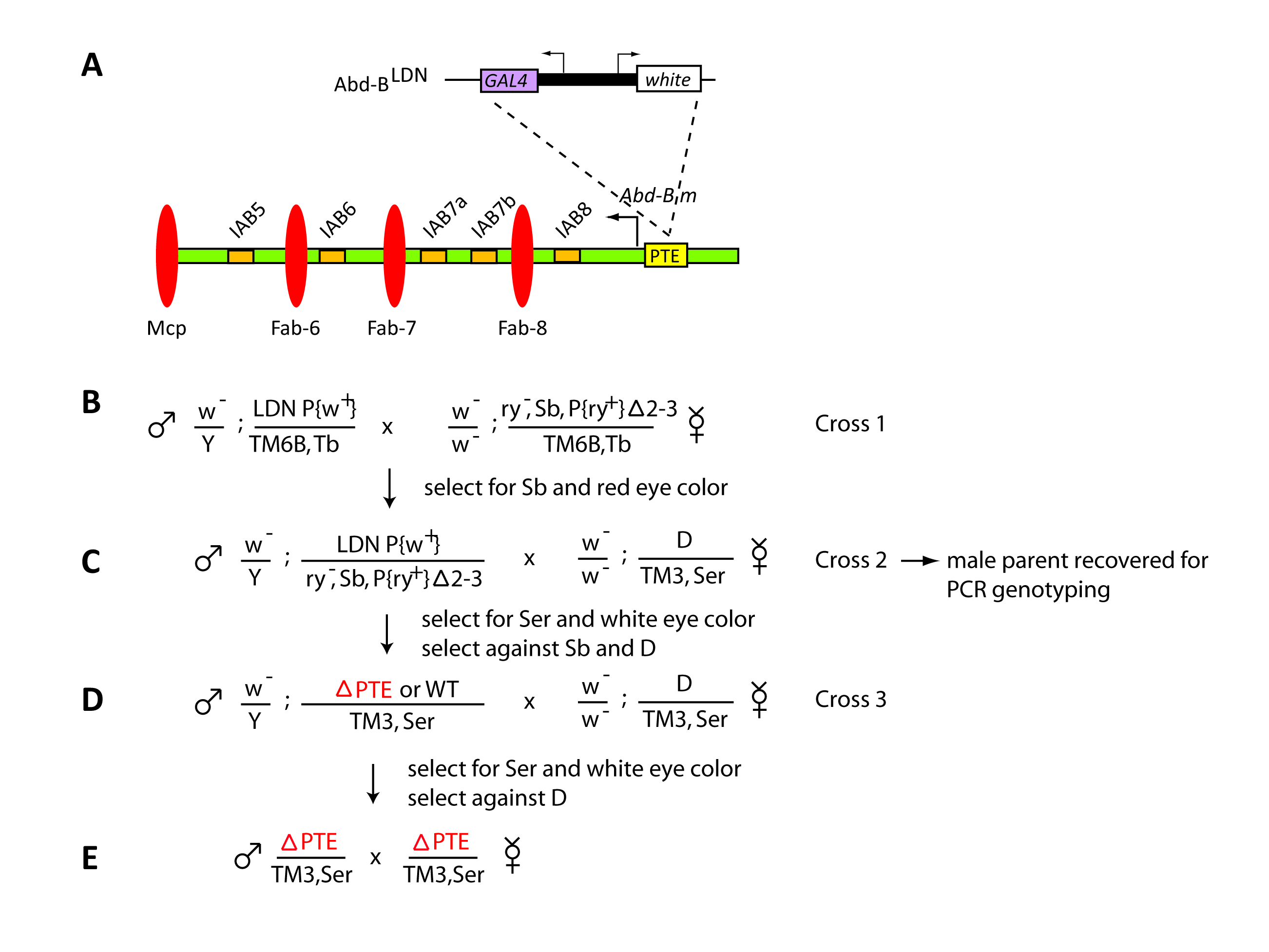

Supplement: Figure S1 — Crosses to generate Abd-BΔPTE-UP mutant. (A) Schematic diagram of the Abd-BLDN P element insertion line, showing the insertion site −241 bp 5′ of the Abd-B m transcription start site in the endogenous PTE sequence. The P element insertion in the Abd-BLDN line contains GAL4 (purple box) and white (white box) reporter genes. The same symbols and color scheme as shown in Fig. 1 are used to show the cis-regulatory modules. (B) Abd-BLDN flies were crossed with a transposase expressing line (Δ2–3) carrying Stubble (Sb) (BL Stock 1798) (Cross 1). Cross 1 male progeny were screened for Sb and variegated red eye color (rather than white eye color). (C) The selected Cross 1 males were crossed with a female with D, a dominant marker, and a TM3 balancer chromosome carrying Serrate (Ser), a dominant phenotypic marker (BL Stock 7198) (Cross 2). Cross 2 male progeny were screened for Ser, white eyes (indicating excision of the GAL4 LDN P element construct), and the absence of Sb and D. (D) The selected Cross 2 male flies were crossed with BL Stock 7198 flies again (Cross 3). After a few days, the male parents were recovered from the Cross 3 vials. PCR amplification of the Abd-B promoter region with primers (black triangles) located 0.5 kb, 1 kb, and 1.5 kb upstream of the Abd-B transcription start site on the genomic DNA prepared from these selected Cross 2 male flies was used to detect deletions in the PTE sequence. Progeny originating from Cross 3 vials seeded with a male parent exhibiting a disruption of the PTE (ΔPTE) were recovered and screened against D and for Ser. (E) These selected Cross 3 progeny were then self-crossed to generate the Abd-B ΔPTE-UP/TM3,Ser balanced line. (TIF) [file pone.0016283.s001.tif]
